# Dietary Lutein and Cognitive Function in Adults: A Meta-Analysis of Randomized Controlled Trials

**DOI:** 10.3390/molecules26195794

**Published:** 2021-09-24

**Authors:** Jeffrey Li, El-Sayed M. Abdel-Aal

**Affiliations:** 1 Department of Nutritional Sciences, University of Toronto, Toronto, ON M5S 1A1, Canada; jeffreyxinyue.li@mail.utoronto.ca; 2 Guelph Research and Development Centre, Agriculture and Agri-Food Canada, Guelph, ON N1G 5C9, Canada

**Keywords:** xanthophyll carotenoid, meta-analysis, adult population, brain health, memory, complex attention, executive function

## Abstract

Emerging literature suggests that dietary lutein may have important functions in cognitive health, but there is not enough data to substantiate its effects in human cognition. The current study was intended to determine the overall effect of lutein on the main domains of cognition in the adult population based on available placebo randomized-controlled trials. Literature searches were conducted in PubMed, AGRICOLA, Scopus, MEDLINE, and EMBASE on 14 November 2020. The effect of lutein on complex attention, executive function and memory domains of cognition were assessed by using an inverse-variance meta-analysis of standardized mean differences (SMD) (Hedge’s *g* method). Dietary lutein was associated with slight improvements in cognitive performance in complex attention (SMD 0.02, 95% CI −0.27 to 0.31), executive function (SMD 0.13, 95% CI −0.26 to 0.51) and memory (SMD 0.03, 95% CI −0.26 to 0.32), but its effect was not significant. Change-from-baseline analysis revealed that lutein consumption could have a role in maintaining cognitive performance in memory and executive function. Although dietary lutein did not significantly improve cognitive performance, the evidence across multiple studies suggests that lutein may nonetheless prevent cognitive decline, especially executive function. More intervention studies are needed to validate the role of lutein in preventing cognitive decline and in promoting brain health.

## 1. Introduction

Lutein and its isomers, zeaxanthin and meso-zeaxanthin, are xanthophyll carotenoids found commonly in green leafy vegetables, avocados and eggs which play significant roles in human health, particularly the health of eyes and brain, due to their antioxidant attributes [1,2,3,4]. Although lutein, unlike β–carotene, does not have vitamin A activity, it is exclusively accumulated in the retina and forms macular pigment [5,6,7,8,9]. Additionally, epidemiological studies have shown that macular pigment optical density (MPOD) status is strongly correlated with lutein intake [1,5,6,7,8,10].

A significant body of literature in the past two decades has evaluated the effect of dietary lutein on human health. Strong evidence has been built on the protective functions of lutein in the eye, particularly in reducing the risk of age-related macular degeneration (AMD) and cataracts [1,4,5,6,9,11,12]. In addition to protecting the retina, lutein has also been observed to preferentially accumulate in the brain across the lifespan [6,13]. Despite lutein comprising only 12% of total carotenoid consumption in infants, it constitutes 60% of the total accumulated carotenoids in the brain. In older adults, lutein constitutes 35% of the accumulated carotenoids in the brain despite only making up 20% of the total plasma carotenoids [6]. Given the exclusive accumulation of lutein in the macula and brain and the connection of the visual to the central nervous system, an emerging body of literature has examined the effect of dietary lutein on cognitive function and brain health.

Cognitive function, in clinical neuropsychology, is conceptualized as several domains of cognition [14,15]. The *Diagnostic and Statistical Manual of Mental Disorders, Fifth Edition* defines these domains as perceptual-motor function, language, executive function, learning and memory, complex attention and social cognition [15]. Cognitive decline is common and normal in ageing populations, with marked decreases in performance across all domains of cognition. Nonetheless, significant loss of function in any domain can cause serious reduction in quality of life marked by early onset of dementia and more serious diseases such as Alzheimer’s [16].

Lutein, as the antioxidant of the brain, is proposed to not only protect cognitive function but also improve cognitive performance [17,18,19,20,21]. Various observational studies have correlated MPOD to greater cognitive health [22,23,24,25,26], whereas others have examined the association between plasma lutein and better cognitive function [21,27,28,29,30,31,32,33,34,35]. Clinical trials have expanded on these results and evaluated whether dietary lutein can improve brain health and cognitive function [18,36,37,38,39,40,41,42,43,44,45,46].

Despite the advancements, there are no conclusive effect sizes for dietary lutein on global cognitive performance nor individual domains of cognition. In addition, there are currently no dietary recommendations for lutein despite its significant roles in human health. The current study was aimed at evaluating the strength of the effect of lutein on improving specific domains of cognitive function in adults. The study was conducted on the adult population since more studies are available in the literature in comparison with infant or adolescent demographic population. To the best of our knowledge, this randomized-controlled trial (RCT) meta-analysis is the first investigation on lutein and cognitive functions.

## 2. Methods

### 2.1. Literature Search and Selection of Studies

**Figure 1:**
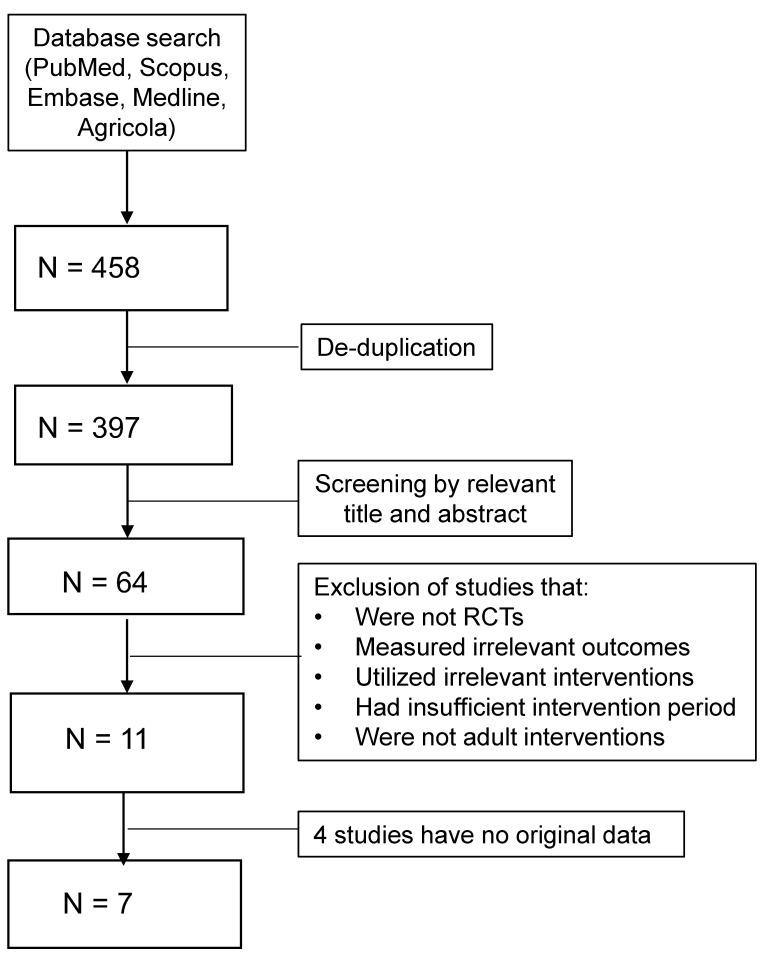
PRISMA flow diagram depicting the number of studies and their exclusion and inclusion in the meta-analysis.

Five databases (PubMed, AGRICOLA, Scopus, EMBASE and MEDLINE) were searched from 2000 to November 2020 for relevant published articles. Each relevant article’s references were also searched for additional publications. The keywords in the search were as follows: (lutein OR zeaxanthin OR macular pigment OR retinal pigment OR xanthophyll carotenoids OR meso-zeaxanthin) AND (Brain OR cognition OR memory OR attention OR language OR executive function OR processing). The searches were limited to randomized-controlled trials, human studies and English publications. All retrieved studies were assessed based on a set of inclusion and exclusion criteria by two reviewers (Figure 1). The main inclusion criteria were studies that investigated cognition health based on cognitive assessments, provided an intervention of dietary lutein in the form of a pill (dietary supplement) or food and examined an adult population. Studies were excluded if they failed to measure specific domains of cognitive performance, are observational, were conducted on children, utilized improper controls and had less than four weeks intervention duration. A dietary lutein intervention could be met by using a lutein supplement, mix of lutein/zeaxanthin/meso-zeaxanthin supplement or a food rich in lutein, along with proper assessments of changes in serum lutein and/or MPOD. Placebo pills or calorically equal meals were acceptable control treatments. Due to the relatively small body of literature evaluating dietary lutein and cognitive function, we did not exclude studies based on their dosage of lutein nor the general age, gender and health status of their populations. Overall, most studies included healthy individuals with consistent gender distribution and ages in the range of 18 to 70 plus.

The total number of studies and their extrusion and inclusion criteria are presented in Figure 1. The initial screening yielded 397 studies, which were cut down to only 64 studies based on their abstracts. Of the 64 studies, 53 were excluded for failing to meet the inclusion/exclusion criteria. The final sample for the meta-analysis comprised of 11 studies, 4 of them failed to provide appropriate data for the meta-analysis. Of the 7 studies included for analysis, 5 treated their subjects with dietary lutein/zeaxanthin/meso-zeaxanthin supplements and the other 2 studies used Hass avocados. Avocados are significant sources of lutein, and both studies evaluated the effectiveness of avocado treatments by observing changes in serum lutein.

### 2.2. Data Extraction and Statistical Analysis

After qualifying for inclusion in the meta-analysis, a set of information that defines each study, e.g., name of the study, year of publication, demographics, type of intervention and dosage (milligrams of lutein per day), type of control, source of intervention, cognitive outcome measurements and duration of the study, was summarized (Table 1). The quality of studies was rated based on the GRADE (Grading of Recommendations, Assessment, Development and Evaluation) approach, and the overall quality of evidence assessment is presented in Table 2. Additionally, the changes from baseline scores were extracted and reported (Table 3). The data for cognitive domains were directly extracted from studies that assessed cognition with a single test. Otherwise, if multiple tests were used to assess a single domain of cognition, the most common test employed across all the studies was used in the meta-analysis. When there were no common tests, the test most utilized in the industry was considered instead. The most common domains and their tests were as follows: memory (Paired Associates Learning-PAL), complex attention (reaction time) and executive function (various tests). Studies that computed a global composite score for cognition but did not report individual scores for each domain were excluded from the analysis (4 studies) (Figure 1).

The R-Studio version 1.4.1103 and version 3.0.1 (R-Studio, Boston, MA, USA) were used to analyze the data. Due to the variety of scales used in assessing cognitive performance, a Hedge’s *g* SMD was calculated for each individual study and inverse-variance pooling was utilized for the overall effect estimate [47]. A separate meta-analysis was conducted for each domain. Given the available data, the following domains were considered: complex attention, executive function and memory. The meta-analysis was conducted using a random-effect model (Sidik–Jonkman or SJ) as interstudy variance disqualified the fixed-effects model. The SJ method was employed over the conventional DerSimonian-Laird (DL) because the DL has been found to be prone to producing false positives, especially when the number of studies is small, and heterogeneity is large [48]. Additionally, the Knapp–Hartung adjustment was also applied along the SJ method as it often outperforms the DL with more robust estimates of the pooled variance [49]. Nonetheless, the Knapp–Hartung–Sidik–Jonkman often produces more conservative estimations with wider confidence intervals [49]. The interstudy heterogeneity was quantified based on the percentage of variation across studies based on *I^2^* statistic and Chi-square parameters. Forest plots were used to display the overall effect size of dietary lutein on cognitive performance for each domain. The overall effect of lutein is presented based on Hedge’s *g* SMD value. For the tests where their lower scores indicate cognitive improvement, the SMD scale was adjusted by multiplying the original mean by −1 to show the improving impact on the positive side for all measurements. Finally, potential publication bias was assessed by using funnel plots assessing the standardized mean difference of each study versus its standard error [50].

## 3. Results

**Figure 2:**
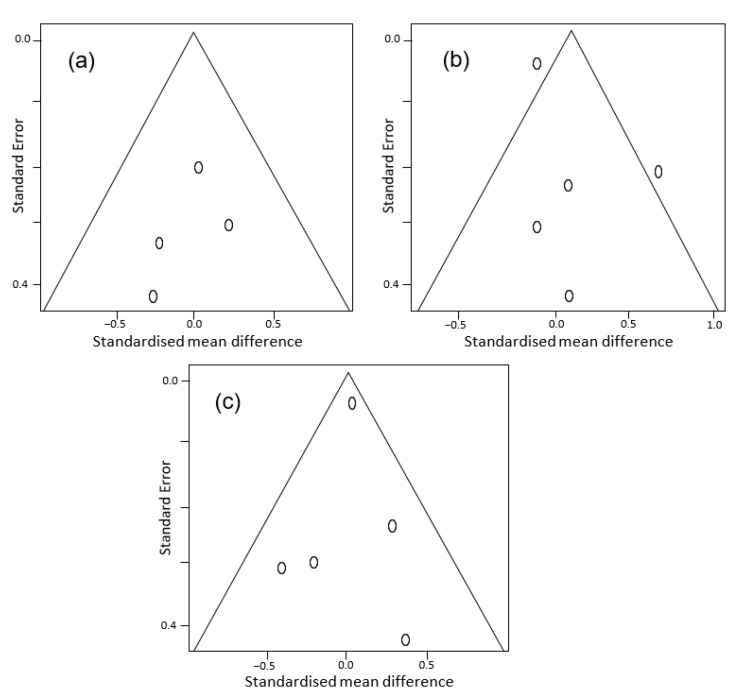
Funnel plots of cognitive domains: (**a**) complex attention, (**b**) executive function and (**c**) memory.

Funnel plots examining publication bias are presented in Figure 2. Overall, there was no publication bias for studies in spite of their small number and relatively high standard error values. For each cognitive domain including complex attention (Figure 2a), executive function (Figure 2b) and memory (Figure 2c), the studies were around the average effect with an acceptable precision being placed inside a symmetric funnel plot except for the executive function domain in two studies. However, the two outlier studies are still acceptable, as they are not far from other studies considering the small number of studies. In addition, the studies examined a variety of health outcomes by using different methods and intervention periods (Table 1). Of the seven studies, five recruited healthy subjects and one recruited overweight and obese patients of the remaining two studies. The second intervention recruited patients with or at risk of age-related macular degeneration. The quality of evidence scores for each domain of cognition are presented in Table 2.

**Figure 3:**
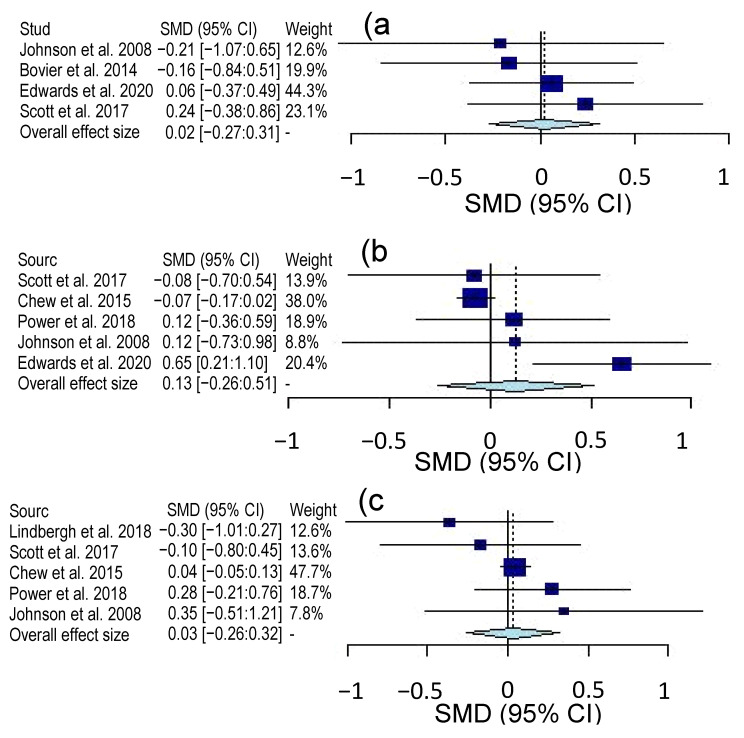
Forest plots of cognitive domains: (**a**) complex attention, (**b**) executive function and (**c**) memory. All plots express effect size as Hedge’s *g* SMD with 95% confidence interval and study weight.

**Figure 4:**
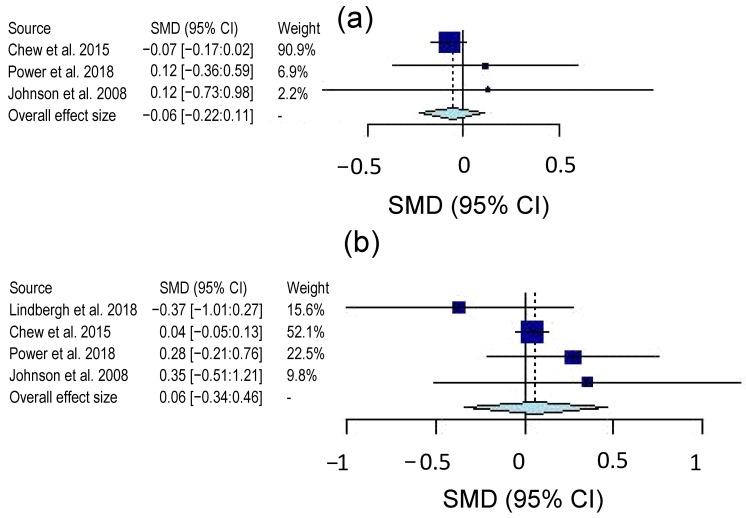
Forest plots of the cognitive domain test scores from lutein supplements only: (**a**) executive function and (**b**) memory. All plots express effect size as Hedge’s *g* SMD with 95% confidence interval and study weight.

Overall, the certainty of evidence for the three cognitive domains was rated as moderate on a 4-level scale (very low, low, moderate and high). Only inconsistency was rated “serious” due to the variety of tools used to assess the three cognitive domains of complex attention, executive function and memory. Table 2 also shows the interstudy heterogeneity for the three cognitive domains based on Chi-square (*X*^2^) and heterogeneity estimate (*I*^2^). The executive function domain showed high values of *X*^2^ and *I*^2^, indicating a high degree of heterogeneity among studies primarily due to the use of various tests and the absence of common or standardized tools to measure executive function. On the other hand, there are at least two common tests for assessing complex attention or memory domain. Forest plots for complex attention, executive function and memory domains are shown in Figure 3. The seven studies were all evaluated, but only four studies assessed the complex attention domain and five interventions measured memory and executive function domains. For complex attention, the Hedge’s *g* value was 0.02 with a 95% confidence interval (CI) of −27:0.31. The Hedge’s *g* value for executive function was 0.13 with a 95% CI of −0.26:0.51 and 0.03 with a 95% CI of −0.26:0.32 for memory domain. As observed in Figure 3, the overall effect of lutein on the three cognitive domains is positioned in the positive side of the plot indicating a beneficial impact for the intervention treatment over the control. The effect of lutein type consumed during the intervention (e.g., supplements or pills versus foods or avocado) was also examined (Figure 4). Only lutein supplement interventions are presented in Figure 4 because the avocado studies produced unmeaningful results due to the small sample size (n = 2). The complex attention domain in pill interventions also showed uninterpretable results due to extremely large confidence intervals. The executive function domain had a *g* value of −0.06 and a 95% CI of −0.22:0.11 (Figure 4a), while the memory domain exhibited a *g* value of 0.06 and a 95% CI of −0.34:0.46 (Figure 4b). Overall, both effect sizes were insignificant, as shown by their respective CIs.

The changes from baseline were retrieved from studies directly if available or were calculated by using the paired sample t-test, and the results are presented in Table 3. There were differences among the studies regarding the effect on the three cognitive domains, which influences the overall effects.

## 4. Discussion

In the current study, we evaluated the overall effect of lutein on the three cognitive domains of complex attention, executive function and memory in adults who have consumed dietary supplements or foods rich in lutein in RCT interventions (Figure 3). The complex attention domain includes processes such as sustained attention, divided attention, selective attention and speed processing, while memory cognitive domain entails functions of free recall, cued recall, recognition memory, long-term memory and implicit learning [15]. The executive function domain is often considered as the most crucial in day-to-day activities such as planning, decision-making, working memory, responding to feedback and flexibility [15]. In the current study, only the adult population was considered in the meta-analysis due to the lack of available data on other demographic groups, especially children. Nonetheless, lutein accumulates over the lifespan, and its functions and benefits are essential over a lifetime [6,9]. Given the function of lutein and other carotenoids such as anti-inflammatory and anti-oxidative agents and their activity throughout the lifespan, the role of lutein is likely based on the protection of cognition and prevention of cognitive decline [4,6,9,20]. In age-related cognitive decline, several domains often begin to underperform simultaneously and a serious loss-of-function in memory, for example, can result in and signal the onset of dementia [16,51]. When cognitive health digresses further, more serious disease such as Alzheimer’s arises. Thus, it is critical to assess the effect of lutein on each domain of cognition in adults in order to better understand its roles not only in preventing cognitive diseases but also in alleviating age-related cognitive decline.

Our results suggest that dietary lutein and its isomers could maintain cognitive functions and brain health, but it did not significantly improve cognitive function in complex attention (Figure 3a), executive function (Figure 3b) or memory (Figure 3c), as indicated by the overall effect size measured by standardized mean difference (SMD). The insignificance of results could be due to the small number of studies and the weight of each study since some of the individual studies show significant effects on cognitive domains. However, in additional analyses, the changes from baseline between intervention and control treatments of individual studies alone revealed that lutein could elicit significant protective functions on cognition and prevent further cognitive decline. Significant improvements from the baseline in individual studies were noted for the executive function in the treatment groups in Scott et al. [38] and Edwards et al. [40] studies (Table 3). In the former study, the treatment group significantly improved in the stockings of Cambridge, a test of spatial planning (*p* = 0.002). In the latter study, the treatment group had similar improvements in the Flanker, a test of response inhibition (*p* < 0.01). For memory, the treatment group of the Lindbergh et al. [44] study did not change significantly from the baseline (*p* = 0.856), whereas the placebo group’s performance showed substantial decline (*p* = 0.084). For complex attention, the treatment group improved from the baseline significantly (*p* < 0.01), whereas the placebo group underwent no significant changes (*p* = 0.91) [46] (Table 3).

Lindbergh et al. [44] reported a control group whose executive function decreases significantly, whereas the lutein treatment group maintains cognitive performance from the baseline. This study was 12 months long and presents a strong case for the protective effect of lutein on brain health. The results of the meta-analysis also suggest that the effect of dietary lutein, although not statistically significant, is most effective on executive function domains in comparison with the other two domains (Figure 3). In the study by Scott et al. [38], the changes from baseline in executive function performance indicates that the control group has experienced no changes, while the avocado group has improved significantly (Table 3) despite the SMD for executive function being −0.08 with a CI from −0.70 to 0.54 (Figure 3). Similarly, the study of Edwards et al. [40] has found executive function improvements in the treatment group from baseline compared with their respective control group (Table 3). Other studies have also reported positive SMDs of 0.12, which indicates consistent beneficial effects of lutein on executive function [39,45]. It is important to consider that the strength of these studies was low due to small sample sizes. In a large trial, Chew et al. [43] found that lutein did not improve cognition, but it may have a positive impact on the prevention of cognitive decline. A crucial consideration must also be made with respect to the Knapp–Hartung–Sidik–Jonkman method of the meta-analysis, as it produces more conservative effect size estimations and larger confidence intervals [45]. Considering the sample size and statistical method applied, there is a high probability that larger trials could demonstrate more benefits of dietary lutein on executive function.

Interestingly, the impact of lutein in the form of pills on executive function was not positive nor statistically significant, as shown in Figure 4a. This is due to the exceptionally high weight of the study of Chew et al. [43] and small sample size of the other two studies [39,45]. The Chew et al. [43] study incorporated two lutein treatment groups in their trial. The first received only lutein and zeaxanthin, whereas the second received the same lutein and zeaxanthin supplement combined with omega-3 fatty acids. They assessed global cognitive performance and found no significant improvements nor differences between the two lutein treatment groups. When their assessment of global cognition is broken down, the effect of lutein on executive function is minimal and slightly negative. On the other hand, the use of lutein in pill form slightly improved memory domain, but it was insignificant (Figure 4b). While the effect of lutein either in pill or food form is most likely dependent on its bioavailability and absorption by different tissues, other factors such as age may have had an impact on the significance of the findings. Given that cognitive performance is closely related to age [43], the supplementation of older populations with lutein may be too late to prevent cognitive decline. The differences among the studies in Figure 4a could be primarily related to the age populations. While Power et al. [45] studied a population with mean age of 45 years, Johnson et al. [39] employed a population with a mean age of 68 years, and Chew et al. [43] studied the oldest population at baseline with mean age of 72 years. These results suggest that the role of lutein in cognition is most likely linked to the retention of cognitive function through ageing in the adult population.

Many studies including those used in the meta-analysis reported improvements in serum lutein [45,46] or MPOD [36,37,38,41,42,44,45] in the treatment group, while only a single study shows insignificant improvement in both measurements, perhaps due to the relative short-term consumption of lutein (3 months) [40]. This could indicate that MPOD accumulated over a long period of time. The consumption of avocado in longer trial durations (6 months) has demonstrated that lutein-rich foods are as effective as lutein supplements in increasing lutein status [38]. This suggests that either lutein supplements or lutein-rich foods could elicit beneficial effects in improving cognitive functions. Research has also shown that lutein bioavailability is enhanced when lutein is consumed with fat [1,2]. The fat intake helps solubilize lutein and resulted in improved absorption. Foods that are high in fat content such as egg yolk have shown higher lutein bioavailability and are more effective in increasing lutein status compared with lutein-rich vegetables such as spinach [1,2]. Similarly, Chew et al. [43] and Johnson et al. [39] incorporated docosahexaenoic acid (DHA) and eicosapentaenoic acid (EPA) (omega-3 fatty acids) in the intervention meals in order to enhance lutein supplementation treatments. These findings emphasize that the addition of fatty acids or fat enhances bioavailability of lutein and eventually its beneficial health effects.

The role of lutein and zeaxanthin in protecting the eye by filtering harmful blue light and maintaining healthy retinal structures and in reducing the risk of ocular diseases such as age-related macular degeneration (AMD) and cataracts has been well documented [1,5,6,11,12,13]. Taking into consideration the protective nature of macular pigments in visual health, macular pigments could also exhibit similar functions in cognition and brain health. Thus, increasing macular pigments via lutein-rich diets and/or dietary supplements will more likely maintain cognition and could reduce the risk of brain diseases. The eye is an extension of the neural system and closely related to the brain and certain cognitive processes. Cortical lutein and zeaxanthin have been hypothesized to have protective functions as the main antioxidants of the brain [17,18,19,20,21]. Although the exact mechanisms of lutein’s neuroprotective effects are still unclear, several mechanisms have been proposed, such as decreased oxidative stress, activation of anti-inflammatory pathways and the modulation of functional properties of synaptic membranes [5,6,13,18]. Furthermore, functional MRI (fMRI) studies have shown that lutein supplementation affects brain morphology and enhances neural response [29,44]. It has been suggested that antioxidants of animal brains restore blood flow following traumatic brain injury and metabolic stress [44]. Comparably, the risk for cerebral hypo-perfusion increases with ageing, which is also associated with cognitive impairment and dementia; therefore, lutein may protect against these effects by increasing blood flow in the brain [44]. All of these studies support the role of lutein and zeaxanthin in brain health and cognitive functions.

Several factors varied among the studies included in the meta-analysis ranging from cognitive performance assessment tools to population characteristics to intervention form and dosage. This undoubtedly affects the outcomes of the meta-analysis, yet the beneficial role of lutein is evident (Figure 3), and significant differences between the treatment and control groups were noticeable in some of the individual studies (Table 3). The use of a large variety of cognitive measurement tools across the studies resulted in an increase in interstudy variations. While the SMD is calculated to correct differences in scale between tests, the SMD cannot adjust for variations in measurement effectiveness of different cognitive tests. Therefore, more standardized tools for cognitive assessment in clinical trials should be established. In addition, the RCTs included in the meta-analysis differed with their subject populations by age, gender and health status, although most were from the USA. Since all the study participants are adults, it becomes difficult to discern whether any significant differences in dietary lutein would be observed between age groups, genders and health statuses. The changes in cognitive performance have been linked with changes in serum lutein and MPOD, although different lutein doses were used in the studies. Currently, no data are available regarding the minimally effective dosage of lutein and its accumulation in brain. Based on the current studies, it appears a minimum intervention length of 4 months is necessary to observe significant changes in lutein status. Thus, further research on the effect of lutein dosage and intervention time would provide insights regarding the role of lutein in cognitive health and its effective dose.

Overall, the meta-analysis based on the available literature indicated that dietary lutein does not consistently improve cognitive function and performance in randomized controlled trials as indicated by the statistically insignificant overall SMD values for the complex attention, executive function and memory domains of cognition. On the other hand, there is evidence in the data from the intra-study treatment/control group changes-from-baseline that dietary lutein may nonetheless be effective in maintaining cognitive function. Thus, large clinical studies with proper designs in terms of intervention duration, dosage and population should be considered to support the role of lutein in brain health.

## 5. Conclusions

The seven RCT studies that met the inclusion criteria have shown insignificant effects for lutein on complex attention, executive function and memory cognitive domains, but slight improvements in the three domains were observed. The studies used different intervention dosages and periods which could influence the overall effect of lutein. Lutein has been found, in many cases, to improve cognitive performance and prevent further cognitive decline. Moreover, dietary lutein affects executive function domain more positively and in terms of greater magnitude than compared to complex attention or memory domain. This suggests that the executive function domain may be more closely related to lutein status, which warrants further investigation and validation. No preference was observed for lutein absorption and accumulation consumed either in dietary pill or food form. Due to the fact that the mechanisms of lutein on neurocognitive processes are unclear, future studies need to consider morphological brain changes in response to dietary lutein over the course of a trial. Additionally, future research should also focus on the possible protective effect of lutein on executive function and other under-evaluated cognitive domains in the literature such as language, social cognition and perceptual-motor function.

## Figures and Tables

**Table 1:** Characteristics of studies included in the meta-analysis.

Study	Population (Age, Location and Health Status)	Treatment (Daily Dose, mg)	Comparison Treatment	Health Outcomes	Intervention Length
Bovier et al. 2014	18–32, USA, Healthy	20 Z & 26 Z + 8 L	Placebo	MPOD, CFF, motor reaction time	4 months
Chew et al. 2015	Mean 72.7, USA, With or at risk of AMD	10 L + 2 Z	Placebo	TICS, MMSE	5 years
Edwards et al. 2020	25–45, USA, BMI >= 27.5	1 avocado (0.5 L)	Isocaloric meal	MPOD, Flanker, Nogo, Oddball	12 weeks
Johnson et al. 2008	60–80, USA, Healthy	12 L & 12 L + 800 DHA	Placebo	Custom tests	4 months
Lindbergh et al. 2017	64–86, USA, Healthy	10 L + 2 Z	Placebo	MPOD, fMRI	12 months
Power et al. 2018	Mean 45.5, USA, Healthy with low MPOD	10 L + 10 meso-Z + 2 Z	Placebo	MPOD, CANTAB	12 months
Scott et al. 2017	>50, USA, Healthy	1 avocado (0.5 L)	Isocaloric meal	MPOD, CANTAB	6 months

Abbreviations: AMD (age-related macular degeneration), CANTAB (Cambridge Neuropsychological test automated battery), CFF (critical flicker fusion), DHA (docosahexaenoic acid), FA (fatty-acid), fMRI (functional magnetic resonance imaging), L (lutein), Z (zeaxanthin), MMSE (mini mental state exam), MPOD (macular pigment optical density) and TICS (telephone interview cognitive status).

**Table 2:** GRADE quality of evidence assessment of complex attention, executive function and memory domains and their heterogeneity assessments.

No of Studies	Certainty Assessment					Participants (n)		Certainty (Overall Quality) ^b^	Heterogeneity (*X*^2^, *I*^2^) ^c^
	Study Design	Risk of Bias ^a^	Inconsistency ^a^	Indirectness ^a^	Imprecision ^a^	Treatment	Control		
Complex attention									
4	RCT	Not serious	Serious	Not serious	Not serious	132	77	Moderate	1.06 (*p* = 0.79), 0% (0–57%)
Executive function									
5	RCT	Not serious	Serious	Not serious	Not serious	1037	1031	Moderate	10.51 (*p* = 0.03), 62% (0–86%)
Memory									
5	RCT	Not serious	Serious	Not serious	Not serious	1018	1007	Moderate	3.42 (*p* = 0.49), 0% (0–76%)

^a^ Scale of 3 levels (not serious, serious and very serious). ^b^ Scale of 4 levels (very low, low, moderate and high). ^c^ *X*^2^ = Chi square, *I*^2^ = heterogeneity statistic.

**Table 3:** Changes from baseline in cognitive scores in comparison with post-intervention.

Study	Measurement	Outcome	Group (n)	Baseline (Mean ± SD)	Post-Intervention (Mean ± SD)	Significance Level
Bovier et al. 2014	Reaction Time (ms)	Complex Attention ^a^	Placebo (10)	219.6 ± 14.2	220.1 ± 20.4	NS
			Treatment (54)	229.9 ± 23.3	223.4 ± 21.6	HS
Chew et al. 2015	Word recall	Memory	Placebo (932)	NA	2.4 ± 2.2	NA
			Treatment (921)	NA	2.5 ± 2.4	NA
	Animal	Executive function	Placebo (933)	NA	16.8 ± 5.4	NA
			Treatment (922)	NA	16.4 ± 5.4	NA
Edwards et al. 2020	Oddball (%)	Complex attention	Placebo (37)	88.6 ± 11.19	92.8 ± 7.6	NS
			Treatment (47)	91.6 ± 7.7	93.2 ± 6.0	NS
	Flanker (%)	Executive function	Placebo	93.5 ± 4.7	92.5 ± 5.9	NS
			Treatment	93.4 ± 5.3	95.6 ± 3.5	HS
Johnson et al. 2008	Pattern recognition Speed (s)	Complex attention ^a^	Placebo (10)	6.8 ± 3.0	5.9 ± 2.3	NS
			Treatment (11)	6.1 ± 2.3	6.4 ± 2.3	NS
	Verbal Fluency	Memory	Placebo	12.9 ± 6.2	13.8 ± 3.5	NS
			Treatment	11.3 ± 5.1	15.5 ± 5.5	S
	Stroop Test (s)	Executive function ^a^	Placebo	25.0 ± 14.8	23.1 ± 22.0	NS
			Treatment	24.2 ± 10.9	21.0 ± 7.8	NS
Lindbergh et al. 2018	Word Recall	Memory	Placebo (14) ^b^	9.4 ± 0.8	8.2 ± 2.3	S
			Treatment (30)	8.9 ± 1.5	8.8 ± 2.2	NS
Power et al. 2018	AST	Executive function	Placebo (31)	841.4 ± 159.0	775.4 ± 217.6	NS
			Treatment (37)	832.0 ± 191.9	751.6 ± 191.7	NS
	PAL (errors)	Memory ^a^	Placebo (31)	4.2 ± 3.8	4.5 ± 4.9	NS
			Treatment (36)	6.8 ± 7.1	3.2 ± 4.5	NS
Scott et al. 2017	CRT (ms)	Complex attention ^a^	Placebo (20)	356.0 ± 70.6	359.1 ± 75.5	NS
			Treatment (20)	347.4 ± 55.4	342.8 ± 56.8	NS
	PAL (errors)	Memory ^a^	Placebo	27.3 ± 18.7	16.8 ± 14.9	NS
			Treatment	28.0 ± 17.8	19.5 ± 15.5	NS
	Stockings of Cambridge (# completed)	Executive function	Placebo	8.0 ± 2.0	9.0 ± 2.7	NS
			Treatment	7.8 ± 2.3	8.8 ± 2.2	HS

^a^ Lower scores are better. ^b^ Placebo group showed a statistical trend toward decline. Abbreviations: AST (attention switching task), CRT (choice reaction time), PAL (paired associates learning), NA (not available), NS (not significant), S (significant, *p* < 0.01) and HS (highly significant, *p* < 0.001).

## Data Availability

Restrictions apply to the availability of these data. Data was obtained from cited research articles and are available from the papers [37,38,39,40,44,45] or upon request from the authors [43].
